# Antibiotic-induced severe cutaneous adverse reactions: a single-center retrospective study over ten years

**DOI:** 10.3389/fimmu.2024.1415830

**Published:** 2024-07-18

**Authors:** Yun Lu, Lu Zhou, Ya Zou, Hua Wei, Yan Zhou, Xirui Guo, Qinchuan Li, Yongqin Ye, Liwen Zhang

**Affiliations:** ^1^ Department of Pharmacy, Chengdu Second People’s Hospital, Chengdu, Sichuan, China; ^2^ Department of Dermatovenereology, Chengdu Second People’s Hospital, Chengdu, Sichuan, China

**Keywords:** antibiotic, retrospective study, severe cutaneous adverse reactions, SJS, TEN, DRESS, AGEP

## Abstract

**Objective:**

Severe cutaneous adverse reactions (SCARs) are rare but life-threatening, with antibiotics being the main cause. This retrospective study from a single center was designed to analyze the culprit drugs, clinical features and treatment outcomes of antibiotic-induced SCARs.

**Methods:**

We analyzed cases of antibiotic-induced SCARs in a tertiary hospital in China between January 2013 and January 2024, including Steven-Johnson syndrome (SJS) or Stevens-Johnson syndrome-toxic epidermal necrolysis (SJS-TEN) overlap, toxic epidermal necrolysis (TEN), drug reaction with eosinophilia and systemic symptoms (DRESS) and acute generalized exanthematous pustulosis (AGEP). Descriptive analysis of the demographic characteristics, clinical manifestations, treatment and prognosis were carried out.

**Results:**

Among 354 cases of SCARs, 63 validated antibiotic-related cases were included. Cephalosporins (31.7%), penicillins (25.4%), and quinolones (19.0%) were the most common triggers for SCARs. Overall, liver (50.8%), lungs (31.7%), and kidneys (23.8%) were the most frequently affected organ in SCARs cases. Eight patients (28.6%) in the SJS/SJS-TEN overlap group and 8 patients (80.0%) in the TEN group received combination therapy of corticosteroids and IVIG. Patients with SCARs caused by penicillins or cephalosporins could receive alternative treatments such as lincomamides, quinolones, and tetracyclines. The mortality rate in the TEN group was the highest at 20.0%, followed by the SJS/SJS-TEN overlap group (7.1%), and no deaths were observed in the DRESS and AGEP groups.

**Conclusion:**

The identification of the culprit antibiotics and the application of alternative antibiotic therapies are crucial for the management of antibiotic-induced SCARs. If complicated underlying conditions and complications like advanced age, cancer and pneumonia coexist with SCARs, patients might be more at risk for mortality.

## 1 Introduction

Severe cutaneous adverse reactions (SCARs) are delayed T-cell-mediated allergic reactions that may be potentially life-threatening (1). The most common SCARs include Steven-Johnson syndrome (SJS), toxic epidermal necrolysis (TEN), drug reaction with eosinophilia and systemic symptoms (DRESS) and acute generalized exanthematous pustulosis (AGEP), with drugs being the main cause of over 85% of SCARs in adults (2, 3). According to reports, the mortality is estimated to range from 10% ~40% for SJS and TEN (4, 5), 2% ~ 10% for DRESS (6, 7), and less than 5% for AGEP (3, 8). For the management of SCARs, it is essential to quickly identify and terminate the culprit drugs.

SJS and TEN are rare and life-threatening SCARs characterized by extensive blisters formation and detachment of the skin with mucosal involvement. The triggering factors of SJS/TEN include drugs, genetic susceptibility, HIV infection, *Mycoplasma pneumoniae* infection, and cancer, etc (9). The respiratory, gastrointestinal, and renal systems are often involved (10). SJS and TEN are variants of the same clinical syndrome, classified into three forms by percentage of body surface epidermal detachment. SJS is defined as detachment encompassing <10% total body surface area (BSA), SJS-TEN overlap involves 10–30% BSA, and TEN involves > 30% BSA (11). The mortality rate of patients with SJS/TEN might be predicted by the severity-of-illness score for toxic epidermal necrolysis (SCORTEN), which is assessed based on 7 independent indicators (12). In the early stage of DRESS, fever (over 38 °C) occurs 2 weeks prior to skin eruption (6, 13). Other typical symptoms of DRESS include lymphadenopathy, extensive rash and facial edema, organ involvement, and hematological disorders, such as eosinophilia and lymphocyte abnormalities (14). The most frequently affected organ is the liver, however, patients with DRESS may also experience involvement of the kidneys, lungs, and heart (15, 16). In addition, AGEP is characterized by numerous small and nonfollicular sterile pustules caused by edematous erythema with few mucosal membrane involvement (3). The most prevalent systemic clinical manifestations were observed to be liver, lung, and renal dysfunctions (17).

The pathogenesis mechanism of SCARs is complex, and different phenotypes are all delayed type IV hypersensitivity reactions (18). Drugs or their metabolites can directly bind covalently with peptides to form new epitopes, known as the “hapten (A) or pro-hapten (B) models”, or directly and non-covalently with the TCR or a peptide-loaded MHC protein known as the “immune receptors (p-i) concept (C) “ (3, 19, 20). They can also indirectly alter the presentation of self-peptides through the pharmacological interaction with the altered peptide repertoire model (D),” resulting in presentation of novel ligands (peptide B), activating autoreactive T cells (3, 20, 21). Additionally, these drug-MHC complexes can activate specific T cell subsets, leading to the release of inflammatory mediators cytokines secreted by natural killer (NK) cells, CD14+CD16+ monocytes, or CD1a + CD14 + dendritic cells and a range of activation signals. These inflammatory mediators further recruit and activate other immune cells, such as macrophages and neutrophils, resulting in intense local inflammation and tissue damage (3, 22).

Antibiotics are the most common culprit drugs that cause immune-mediated drug reactions, including allergic reactions, organ specificity, and SCARs, which pose an undeniable threat to patient safety and public health (23). Although antibiotics are recognized as the most common causative drugs for SCARs (24), minimal research is available on long-term systemic SCARs specifically caused by antibiotics. Owing to the widespread usage of antibiotics and their high risk of inducing SCARs, more comprehensive research on antibiotic-induced SCARs is still needed to better understand the epidemiology and culprit drugs in different regions and populations. In this study, we analyzed the causative antibiotics, demographic characteristics, clinical manifestations, treatment and prognosis of patients with SCARs from a single center over a ten-year period. The results may provide valuable references for the early prevention, diagnosis, and later treatment of SCARs, thereby contributing to the effective management of antibiotic-induced SCARs.

## 2 Materials and methods

### 2.1 Data sources and case selection

We retrospectively reviewed the medical records of patients diagnosed with SCARs, including SJS/TEN, DRESS, and AGEP in Institute of Dermatology, Chengdu Second People’s Hospital, Sichuan, China between January 2013 and January 2024. Diagnosis of SJS/TEN was based on consensus definition and clinical characteristics. Patients with acute onset of mucous membrane involvement, extensive macules and blisters with skin detachment of less than 10% of BSA are classified as SJS. Skin detachment greater than 30% of BSA is considered TEN, whereas between 10% and 30% is categorized as SJS-TEN overlap (25). Diagnosis of DRESS was based on the criteria proposed by the European Registry of Severe Cutaneous Adverse Reaction (RegiSCAR) (26). Patients with at least three of the following criteria were recruited in this study: suspected drug reaction with an acute skin rash, fever (>38°C), enlarged lymph nodes at least two sites, internal organ involvement and hematologic abnormalities (27). Diagnosis of AGEP was according to the RegiSCAR criteria, and the AGEP validation score involves the histology findings, clinical course and morphology (28).

**Figure 1:**
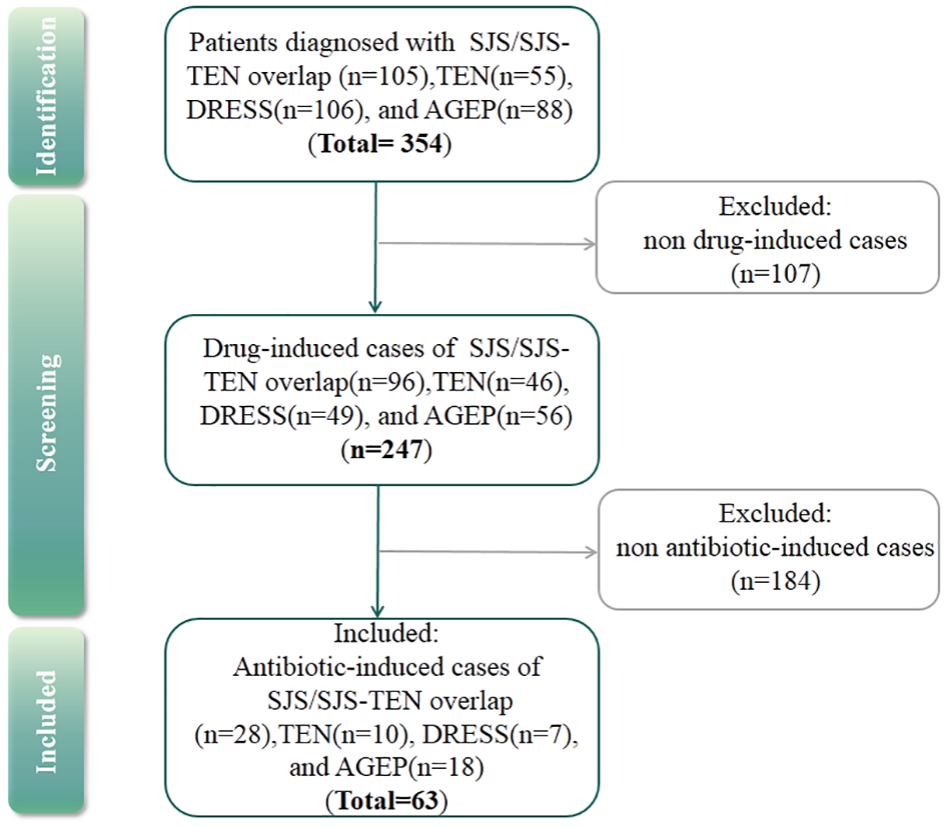
Flow chart for case selection in this study.

According to the admission records of each patient, antibiotic-induced SCARs were included in the study, and others were excluded (**Figure 1**). Drug causality was evaluated using the ALDEN score for SJS/TEN and the Naranjo score for DRESS and AGEP. Drugs with ALDEN scores<2 (SJS/TEN) or Naranjo algorithm scores<1 (DRESS and AGEP) were excluded. Due to the high risk of false causal inference, corticosteroids were excluded, which could be used for the treatment of SCARs (29). Then, the drug with the highest score was considered the culprit drug. If multiple antibiotics had the same score, they were all considered the culprit drugs. However, if antibiotics were used in combination with other drugs owing the same score, then single drug causality was not assigned. The study was approved by the ethical committee of the institution.

### 2.2 Clinical characteristics analysis

Detailed information was collected, which included age, sex, latency, drug allergy history, immunocompromised status, underlying disease, causative antibiotics, secondary skin infections and mucocutaneous lesions, organ involvement and complications, hematologic abnormalities, treatment, length of hospital stay and mortality. Drug causality of SJS/TEN was assessed using the ALDEN score that includes very unlikely (<0), unlikely (0–1), possible (2-3), probable (4-5) and very probable (≥6) (2). Meanwhile, drug causality of DRESS and AGEP was assessed by the Naranjo score, which is as follows: doubtful (≤0), possible (1-4), probable (5-8), and definite (≥9) (30). To evaluate the prognosis of patients with SJS/TEN, mortality was predicted using the SCORTEN standard system, which was evaluated based on 7 clinical indicators (12).

### 2.3 Statistical analysis

Descriptive statistics were conducted. Statistical analysis was performed using SPSS software, version 23.0. Continuous data were represented by the median (interquartile range [IQR]), whereas categorical variables were described by frequency (%). Comparisons of categorical variables among groups were analyzed using Fisher’s exact test. Continuous variables were analyzed using one-way ANOVA or Mann–Whitney U test. *P <*0.05 was considered to indicate a significant difference.

## 3 Results

### 3.1 Demographic characteristics

**Figure 2:**
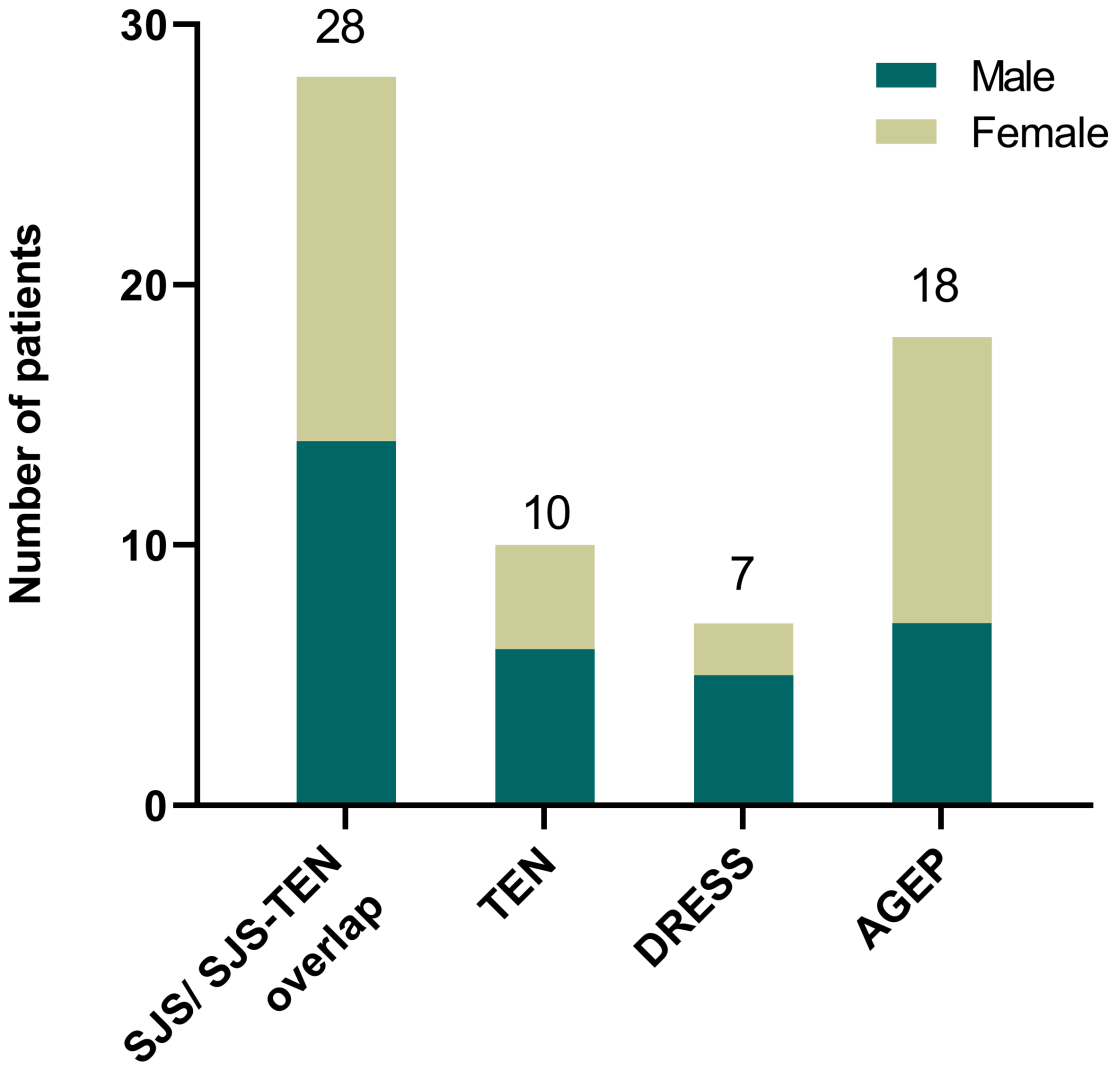
Type of antibiotic-induced SCARs in this study.

Out of the 354 SCARs cases, 107 cases caused by infections, autoimmune diseases such as systemic lupus erythematosus or with undefined causes were excluded. Then, among 247 drug-induced SCAR cases, 184 non antibiotic-induced cases were further excluded (**Figure 1**). As a result, a total of 63 patients with antibiotic-induced SCARs were included in the study, comprising 28 (44.4%) cases of SJS or SJS-TEN overlap, 10 (15.9%) of TEN, 7 (11.1%) of DRESS, and 18 (28.6%) of AGEP. The demographic characteristics are shown in **Table 1**. Median age was highest for TEN (63.5 years, IQR 49.5-68.5), followed by DRESS (58.0 years, IQR 54.0-68.5), SJS/SJS-TEN overlap (43.5 years, IQR 29.3-61.3) and AGEP (25.5 years, IQR 18.0-31.0) (*P*<0.001). The male to female ratios of the SJS/SJS-TEN overlap, TEN, DRESS and AGEP groups were 1:1, 1.5:1, 2.5:1, and 1:1.6, respectively (**Figure 2**). Among patients with a fever (≥38.5°C) during hospitalization, TEN possessed the highest proportion (50.0%), followed by SJS/SJS-TEN overlap (14.3%), DRESS (14.3%), and AGEP (5.6%) (**Table 1**). Additionally, the incubation period from the first drug intake to the onset of symptoms varied throughout the groups. In the DRESS group, the median latent period of antibiotic exposure (7.0 days, IQR 3.0-8.5) was the longest, while TEN showed the shortest latency of 1.5 days (IQR 1.0-2.0) (*P*<0.001) (**Table 1**).

**Table 1:** Demographic data of the patients with antibiotic-induced SCARs.

Characteristics		Phenotypes, No. (%)				*P* value
	Total (n=63)	SJS or SJS-TEN overlap (n=28)	TEN (n=10)	DRESS (n=7)	AGEP (n=18)	
Age, y, median (IQR)	43.0 (27.0-61.5)	43.5 (29.3-61.3)	63.5 (49.5-68.5)	58.0 (54.0-68.5)	25.5 (18.0-31.0)	**<0.001**
Sex						
Male	32 (50.8)	14 (50.0)	6 (60.0)	5 (71.4)	7 (38.9)	0.492
Female	31 (49.2)	14 (50.0)	4 (40.0)	2 (28.6)	11 (61.1)	0.492
Fever	18 (28.6)	7 (25.0)	5 (50.0)	3 (42.9)	3 (16.7)	0.226
≥38.5°C	11 (17.4)	4 (14.3)	5 (50.0)	1 (14.3)	1 (5.6)	**0.035**
Latency, d, median (IQR)	3.0 (2.0-5.0)	3.0 (2.0-5.0)	1.5 (1.0-2.0)	7.0 (3.0-8.5)	3.0 (2.0-5.0)	**<0.001**
0-7	54 (85.7)	23 (82.1)	10 (100.0)	5 (71.4)	16 (88.9)	0.353
8-24	9 (14.3)	5 (17.9)	0 (0.0)	2 (28.6)	2 (11.1)	0.353
Underlying disease						
Cancer	4 (6.3)	1 (3.6)	1 (10.0)	2 (28.6)	0 (0.0)	**0.062**
Other autoimmune disease	5 (7.9)	0 (0.0)	2 (20.0)	2 (28.6)	1 (5.6)	**0.013**
Diabetes	6 (9.5)	3 (10.7)	1 (10.0)	2 (28.6)	0 (0.0)	0.130
Hypertension	11 (17.4)	7 (25.0)	2 (20.0)	2 (28.6)	0 (0.0)	0.065
Liver disease	5 (7.9)	1 (3.6)	2 (20.0)	1 (14.3)	1 (5.6)	0.251
Chronic kidney disease	3 (4.8)	1 (3.6)	2 (20.0)	0 (0.0)	0 (0.0)	0.173
Lung disease	12 (19.0)	4 (14.3)	4 (40.0)	3 (42.9)	1 (5.6)	**0.041**
Heart disease	4 (6.3)	3 (10.7)	1 (10.0)	0 (0.0)	0 (0.0)	0.479
Prior antibiotic allergies, n (%)	13 (20.6)	7 (25.0)	2 (20.0)	1 (14.3)	3 (16.7)	0.965
Length of hospital stay after onset of SCARs, d, median (IQR)	12.0 (8.0-19.0)	13.0 (9.0-18.0)	24.0 (20.0-29.5)	14.0 (9.5-20.0)	8.0 (7.0-12.0)	**<0.001**

The bold values represent statistically significant P-values (P<0.05).

Among the 63 SCARs cases, 27 patients (42.9%) were found to have at least one potential underlying disease (**Table 1**). In general, lung disease (n=12, 19.0%), hypertension (n=11, 17.5%), and diabetes (n=6, 9.5%) were the most common underlying disorders among these SCARs cases. Notably, cancer was present in 4 cases, including 1 case (3.6%) of SJS, 1 case (10.0%) of TEN, and 2 cases (28.6%) of DRESS (**Table 1**). Thirteen patients showed history of antibiotic allergies, including cephalosporins (2 SJS/SJS-TEN overlap, 2 TEN, 2 AGEP and 1 DRESS), penicillins (3 SJS/SJS-TEN overlap, 1 TEN, and 1 AGEP), levofloxacin (1 TEN and 1 AGEP), and metronidazole (1 SJS/SJS-TEN overlap) (**Supplementary Table S1**). Among them, 1 patient with TEN was allergic to both cephalosporins and penicillins, while 1 patient with AGEP showed allergy to both cephalosporins and levofloxacin (**Supplementary Table S1**). However, there was no significant difference in the allergy history among different groups (*P*=0.965, **Table 1**). According to the length of hospital stay after onset of SCARs, patients with TEN seemed to have the longest median hospitalization time of 24.0 days (IQR 20.0-29.5) (*P*<0.001, **Table 1**).

### 3.2 Causative antibiotics

**Figure 3:**
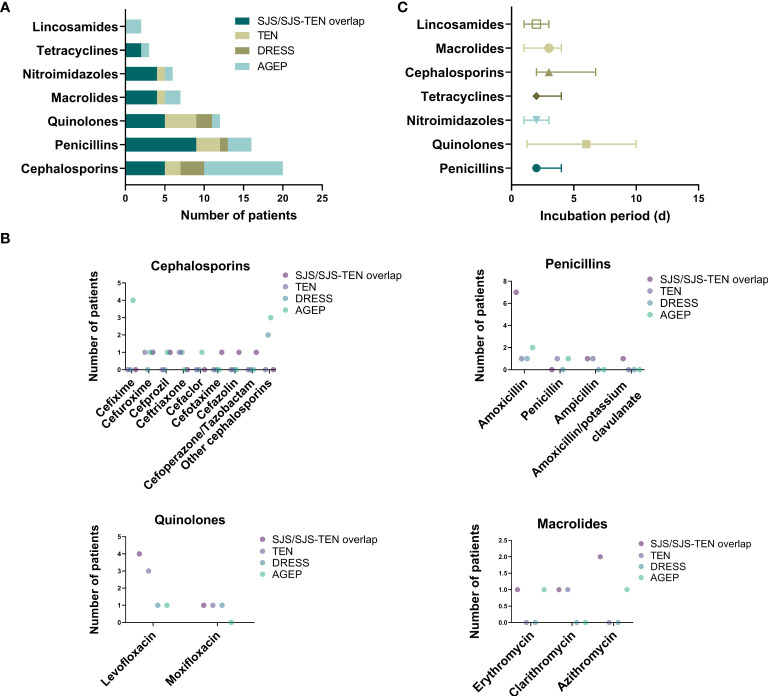
Culprit antibiotics of patients with SCARs. **(A)** Case number of SCARs triggered by different antibiotics; **(B)** Case number of SCARs triggered by specific drugs in different groups; **(C)** Incubation period of culprit antibiotics.

As for causative drugs, 7 classes of culprit antibiotics were recorded (**Table 2**), including cephalosporins, penicillins, quinolones, macrolides, nitroimidazoles, tetracyclines, and lincosamides. Notably, as shown in **Supplementary Table S2**, most antibiotics were used for treatment of cold (38.1%), with others involving toothache (11.1%), pharyngitis (7.9%), and pulmonary infections (7.9%), etc. Overall, cephalosporins (n=20, 31.7%), penicillins (n=16, 25.4%), and quinolones (n=12, 19.0%) were the most commonly implicated drugs of SCARs (**Figures 3A, B**). Other causative drugs also included macrolides (n=7, 11.1%), nitroimidazoles (n=6, 9.5%), tetracyclines (n=3, 4.8%), and lincosamides (n=2, 3.2%). However, there seemed to be a significant difference in the cases of different types of SCARs caused only by cephalosporins (*P*=0.041, **Table 2**). For patients with SJS/SJS-TEN overlap, penicillins were found to be the most culpable drugs in 9 cases (32.1%), followed by cephalosporins (n=5, 17.9%), and quinolones (n=5, 17.9%) (**Table 2**). In patients with TEN, quinolones (n=4, 40.0%), penicillins (n=3, 30.0%), and cephalosporins (n=2, 20.0%) were the most frequent causative antibiotics (**Table 2**). However, for the DRESS group, cephalosporins and quinolones were considered the culprit drugs, accounting for 42.9% and 28.6%, respectively. Among patients with AGEP, cephalosporins (n=10, 55.6%) were the main triggers, followed by penicillins (n=3, 16.7%) (**Table 2**). Specifically, amoxicillin (17.5%), levofloxacin (14.3%), metronidazole (7.9%), and cefixime (6.3%) seemed to be the most frequent culprit drugs (**Table 2**). The drug latency period varied among different groups of culprit antibiotics, and quinolones showed the longest median latent period of 6.0 days (IQR 1.8-10.0) (**Figure 3C**).

**Table 2:** Causative drugs of SCARs.

Culprit Drugs	Incubation period (d), median (IQR)		No. (%) of cases				*P* value
		Total (n=63)	SJS or SJS-TEN overlap (n=28)	TEN (n=10)	DRESS (n=7)	AGEP (n=18)	
**Cephalosporins**	**3.0 (2.0-6.3)**	**20 (31.7)**	**5 (17.9)**	**2 (20.0)**	**3 (42.9)**	**10 (55.6)**	**0.041**
Cefixime	7.5 (6.0-9.0)	4 (6.3)	0 (0.0)	0 (0.0)	0 (0.0)	4 (22.2)	
Cefuroxime	3.0 (2.5-3.0)	3 (4.8)	1 (3.6)	1 (10.0)	0 (0.0)	1 (5.6)	
Cefprozil	3.5 (2.3-4.8)	2 (3.2)	1 (3.6)	0 (0.0)	0 (0.0)	1 (5.6)	
Ceftriaxone	6.0 (4.0-8.0)	2 (3.2)	0 (0.0)	1 (10.0)	1 (14.3)	0 (0.0)	
Cefaclor	5	1 (1.6)	0 (0.0)	0 (0.0)	0 (0.0)	1 (5.6)	
Cefotaxime	3	1 (1.6)	1 (3.6)	0 (0.0)	0 (0.0)	0 (0.0)	
Cefazolin	5	1 (1.6)	1 (3.6)	0 (0.0)	0 (0.0)	0 (0.0)	
Cefoperazone/ Tazobactam	21	1 (1.6)	1 (3.6)	0 (0.0)	0 (0.0)	0 (0.0)	
Other cephalosporins	2.0 (2.0-2.0)	5 (7.9)	0 (0.0)	0 (0.0)	2 (28.6)	3 (16.7)	
**Penicillins**	**2.0 (2.0-3.5)**	**16 (25.4)**	**9 (32.1)**	**3 (30.0)**	**1 (14.3)**	**3 (16.7)**	0.580
Amoxicillin	2.0 (2.0-4.5)	11 (17.5)	7 (25.0)	1 (10.0)	1 (14.3)	2 (11.1)	
Penicillin	2.0 (2.0-2.5)	2 (3.2)	0 (0.0)	1 (10.0)	0 (0.0)	1 (5.6)	
Ampicillin	1	2 (3.2)	1 (3.6)	1 (10.0)	0 (0.0)	0 (0.0)	
Amoxicillin/ potassium clavulanate	2	1 (1.6)	1 (3.6)	0 (0.0)	0 (0.0)	0 (0.0)	
**Quinolones**	**6.0 (1.8-10.0)**	**12 (19.0)**	**5 (17.9)**	**4 (40.0)**	**2 (28.6)**	**1 (5.6)**	0.119
Levofloxacin	2.0 (1.0-10.0)	9 (14.3)	4 (14.3)	3 (30.0)	1 (14.3)	1 (5.6)	
Moxifloxacin	3.5 (2.0-6.5)	3 (4.8)	1 (3.6)	1 (10.0)	1 (14.3)	0 (0.0)	
**Macrolides**	**3.0 (1.0-3.5)**	**7 (11.1)**	**4 (14.3)**	**1 (10.0)**	**0 (0.0)**	**2 (11.1)**	0.937
Erythromycin	1.0 (1.0-1.0)	2 (3.2)	1 (3.6)	0 (0.0)	0 (0.0)	1 (5.6)	
Clarithromycin	2.0 (1.0-2.5)	2 (3.2)	1 (3.6)	1 (10.0)	0 (0.0)	0 (0.0)	
Azithromycin	4.0 (3.5-7.0)	3 (4.8)	2 (7.1)	0 (0.0)	0 (0.0)	1 (5.6)	
**Nitroimidazoles **	**2.0 (1.3-2.8)**	**6 (9.5)**	**4 (14.3)**	**1 (10.0)**	**0 (0.0)**	**1 (5.6)**	0.806
Metronidazole	2.0 (1.0-3.0)	5 (7.9)	4 (14.3)	1 (10.0)	0 (0.0)	0 (0.0)	
Tinidazole	2	1 (1.6)	0 (0.0)	0 (0.0)	0 (0.0)	1 (5.6)	
**Tetracyclines**	**2.0 (2.0-3.0)**	**3 (4.8)**	**2 (7.1)**	**0 (0.0)**	**0 (0.0)**	**1 (5.6)**	1.000
Doxycycline	4	1 (1.6)	1 (3.6)	0 (0.0)	0 (0.0)	0 (0.0)	
Minocycline	2	1 (1.6)	0 (0.0)	0 (0.0)	0 (0.0)	1 (5.6)	
Oxytetracycline	2	1 (1.6)	1 (3.6)	0 (0.0)	0 (0.0)	0 (0.0)	
**Lincosamides**	**2.0 (1.5-2.5)**	**2 (3.2)**	**0 (0.0)**	**0 (0.0)**	**0 (0.0)**	**2 (11.1)**	0.160
Clindamycin	2.0 (1.5-2.5)	2 (3.2)	0 (0.0)	0 (0.0)	0 (0.0)	2 (11.1)	
**Multiple drugs**	**5.0(4.0-6.0)**	**3(4.8)**	**1(3.6)**	**0(0.0)**	**2(28.6)**	**0(0.0)**	**0.017**

The bold values represent the values of antibiotic categories.

### 3.3 Clinical features and complications

As one of the typical characteristics of SJS/TEN, the mucosa in the SJS/TEN groups was most severely involved (*P*=0.001), which mainly included the lips and oral mucosa (SJS/SJS-TEN overlap, 96.4%; TEN, 80.0%), ocular mucosa (SJS/SJS-TEN overlap, 53.6%; TEN, 60.0%), and genital mucosa (SJS/SJS-TEN overlap, 46.4%; TEN, 50.0%) (**Table 3**). A few cases also included involvement of nasal (4.8%) and anal (3.2%) mucosa. In addition, the involvement of at least 3 mucosal areas accounted for 36.8% in the SJS/TEN group. However, mucosal involvement was rarely observed in DRESS and AGEP cases (**Table 3**). In addition, the rate of secondary skin infections in the TEN group was as high as 70.0%, significantly higher than other groups (*P*=0.013).

**Table 3:** Clinical characteristics of patients with antibiotic-related SCARs.

Characteristics		No. (%) of cases				*P* value
	Total (n=63)	SJS or SJS-TEN overlap (n=28)	TEN (n=10)	DRESS (n=7)	AGEP (n=18)	
Mucosal involvement						
Ocular mucosa	23 (36.5)	15 (53.6)	6 (60.0)	1 (14.3)	1 (5.6)	**0.001**
Lip and Oral mucosa	38 (60.3)	27 (96.4)	8 (80.0)	1 (14.3)	2 (11.1)	**<0.001**
Nasal mucosa	3 (4.8)	3 (10.7)	0 (0.0)	0 (0.0)	0 (0.0)	0.410
Genital mucosa	21 (33.3)	13 (46.4)	5 (50.0)	1 (14.3)	2 (11.1)	**0.031**
Anal mucosa	2 (3.2)	1 (3.6)	1 (10.0)	0 (0.0)	0 (0.0)	0.548
Involvement of mucosal areas (≥3)	15 (23.8)	9 (32.1)	5 (50.0)	1 (14.3)	0 (0.0)	**0.005**
Secondary skin infections	22 (34.9)	11 (39.3)	7 (70.0)	0 (0.0)	4 (22.2)	**0.013**
Lymphadenopathy	8 (12.7)	0 (0.0)	0 (0.0)	5 (71.4)	3 (16.7)	**<0.001**
Pneumonia	20 (31.7)	7 (25.0)	7 (70.0)	4 (57.1)	2 (11.1)	**<0.001**
Liver dysfunction	32 (50.8)	13 (46.4)	8 (80.0)	6 (85.7)	5 (27.8)	**0.012**
Renal dysfunction	15 (23.8)	7 (25.0)	6 (60.0)	3 (42.9)	1 (5.6)	**0.005**
GI bleeding	9 (14.3)	3 (10.7)	2 (20.0)	2 (28.6)	2 (11.1)	0.535
Heart involvement	14 (22.2)	6 (21.4)	5 (50.0)	2 (28.6)	1 (5.6)	**0.045**
Hypoproteinemia	21 (33.3)	7 (25.0)	7 (70.0)	5 (71.4)	2 (11.1)	**0.001**
Sepsis	3 (4.8)	1 (3.6)	2 (20.0)	0 (0.0)	0 (0.0)	0.171
Hematologic abnormalities at admission day, median (IQR)						
Eosinophils count	/	0.03 (0.01-0.12)	0.00 (0.00-0.01)	2.0 (1.2-5.4)	0.27 (0.12-0.54)	**0.002**
Platelets count	/	249.0 (205.5-302.8)	144.0 (120.5-217.3)	199.0 (168.0-245.5)	256.0 (229.8-294.8)	**<0.001**
Lymphocytes count	/	1.7 (1.0-2.5)	0.7 (0.4-0.9)	2.4 (1.5-3.3)	1.8 (1.2-2.4)	**<0.001**
White blood cells count	/	9.3 (6.2-11.7)	6.2 (3.5-9.1)	14.6 (12.2-26.9)	11.1 (9.3-13.1)	**<0.001**
Neutrophils count	/	6.3 (4.6-8.5)	4.2 (2.3-8.4)	7.5 (6.5-13.8)	7.4 (5.5-10.4)	**<0.001**
Heart rate at admission day (/min)	/	79.0 (76.0-87.0)	82.5 (72.8-88.0)	78.0 (78.0-79.0)	80.0 (78.3-83.5)	**<0.001**
Serum BUN at admission day (mg/dL)	/	5.3 (4.1-8.2)	8.2 (6.9-14.2)	4.9 (4.1-6.2)	3.6 (3.1-4.4)	**<0.001**
Serum glucose at admission day (mg/dL)	/	6.5 (4.6-7.6)	6.0 (5.5-7.7)	5.6 (5.0-7.0)	5.0 (4.6-5.9)	**<0.001**

The bold values represent statistically significant P-values (P<0.05).

Overall, liver (50.8%) was the most frequently affected organ in SCARs cases, followed by the lungs (31.7%), kidneys (23.8%), heart involvement (22.2%), and Gastrointestinal (GI) bleeding (14.3%) (**Table 3**). For SJS/TEN group, the most common complications included liver dysfunction (SJS/SJS-TEN overlap, 46.4%; TEN, 80.0%), pneumonia (SJS/SJS-TEN overlap, 25.0%; TEN, 70.0%), and renal dysfunction (SJS/SJS-TEN overlap, 25.0%; TEN, 60.0%) (**Table 3**). One of the features of the DRESS group was a high percentage of lymphadenopathy—up to 71.4%. Moreover, liver dysfunction (85.7%), pneumonia (57.1%), and renal dysfunction (42.9%) were also frequently observed (**Table 3**). However, the AGEP group had a comparatively low tendency for severe complications, with the most common being liver dysfunction (27.8%), lymphadenopathy (16.7%), pneumonia (11.1%), and GI bleeding (11.1%) (**Table 3**). In addition, the DRESS group was more prone to hematological abnormalities, such as significantly higher median counts of eosinophils, lymphocytes, white blood cells, and neutrophils, than the other groups (*P*<0.05, **Table 3**). In addition, the TEN and DRESS groups showed lower platelet counts compared to the other two groups (*P*<0.001, **Table 3**). Heart rate and serum glucose at admission day was the highest in the TEN group, while serum glucose at admission day was the highest in the SJS/SJS-TEN overlap group (*P*<0.001, **Table 3**).

### 3.4 Treatment and outcome

**Figure 4:**
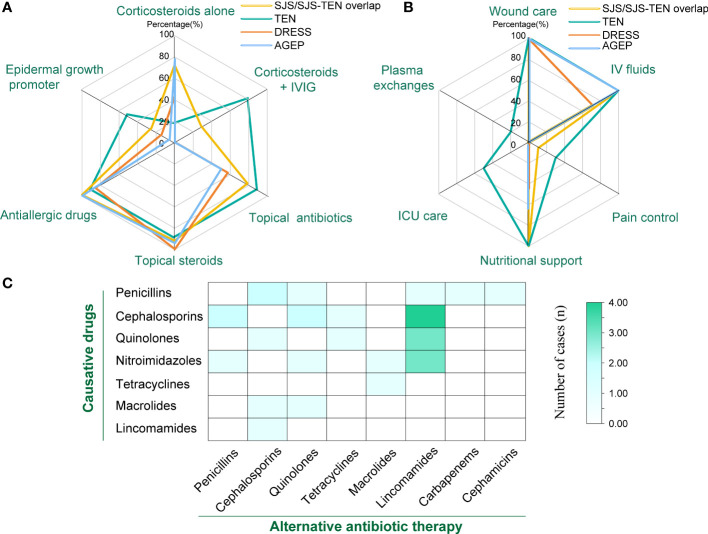
Therapy for treatment of SCARs. **(A)** Number of patients treated with corticosteroids, IVIG, and other medications in different groups; **(B)** Number of patients receiving supportive care, ICU care, and plasma exchange in different groups; **(C)** Heat map of the number of cases with different classes of causative drugs and corresponding antibiotic replacement therapy.

The treatments for patients with SCARs included corticosteroids, intravenous immunoglobulin (IVIG), other medication therapies, and supportive care (**Table 4**). A total of 28.6% (n=8) patients in the SJS/SJS-TEN overlap received combined application of corticosteroids and IVIG, while up to 80.0% (n=8) of patients in the TEN group received this therapy (**Figure 4A**, **Table 4**). However, partial patients with DRESS (42.9%, n=3) and AGEP (77.8%, n=14) received systemic corticosteroid therapy, while neither of them received IVIG treatment (**Figure 4A**). In addition, 69.8% of the SCARs patients received topical antibiotics to improve skin infection status, and 96.8% of the patients were treated with antiallergic drugs like ebastine, levocetirizine, olopatadine and loratadine (**Table 4**). The vast majority of patients (93.6%) used topical steroids such as desonide and halometasone to improve skin inflammation (**Table 4**). Recombinant human epidermal growth factor gel was employed to improve skin damage repair for partial patients (n=14, 22.2%), with TEN patients (n=5, 50.0%) accounting for the highest (*P*=0.048, **Figure 4A**). In particular, 4 SCARs patients (2 DRESS, 2 AGEP) received cyclosporine treatment, and 1 AGEP patient received methotrexate treatment (**Table 4**). For supportive care (**Table 4**, **Figure 4B**), all patients of SCARs (100.0%) received wound care and 96.8% received IV fluids. Six patients of SJS/TEN were also treated with pain control. In particular, 5 TEN patients with severe complications were supported by the ICU care (*P*<0.001), of which 2 patients underwent plasma exchanges for 3-5 times due to their critical conditions (**Table 4**).

**Table 4:** Treatment and prognosis of patients with antibiotic-related SCARs.

Treatment and prognosis		No. (%) of cases				*P* value
	Total (n=63)	SJS or SJS-TEN overlap (n=28)	TEN (n=10)	DRESS (n=7)	AGEP (n=18)	
Corticosteroids alone	39 (61.9)	20 (71.4)	2 (20.0)	3 (42.9)	14 (77.8)	**0.009**
Corticosteroids + IVIG	16 (25.4)	8 (28.6)	8 (80.0)	0 (0.0)	0 (0.0)	**<0.001**
Topical antibiotics	44 (69.8)	22 (78.6)	9 (90.0)	4 (57.1)	9 (50.0)	0.079
Topical steroids	59 (93.6)	26 (92.9)	9 (90.0)	7 (100.0)	17 (94.4)	1.000
Antiallergic drugs	61 (96.8)	28 (100.0)	9 (90.0)	6 (85.7)	18 (100.0)	0.070
Epidermal growth promoter	14 (22.2)	7 (25.0)	5 (50.0)	1 (14.3)	1 (5.6)	**0.048**
Cyclosporin	4 (6.3)	0 (0.0)	0 (0.0)	2 (28.6)	2 (11.1)	**0.021**
Methotrexate	1 (1.6)	0 (0.0)	0 (0.0)	0 (0.0)	1 (5.6)	0.556
Alternative antibiotics	27 (42.9)	14 (50.0)	5 (50.0)	3 (42.9)	5 (27.8)	0.514
Supportive care						
Wound care	63 (100.0)	28 (100.0)	10 (100.0)	7 (100.0)	18 (100.0)	/
IV fluids	61 (96.8)	28 (100.0)	10 (100.0)	5 (71.4)	18 (100.0)	**0.011**
Pain control	6 (9.5)	3 (10.7)	3 (30.0)	0 (0.0)	0 (0.0)	0.066
Nutritional support	52 (82.5)	28 (100.0)	10 (100.0)	3 (42.9)	11 (61.1)	**<0.001**
ICU care	5 (7.9)	0 (0.0)	5 (50.0)	0 (0.0)	0 (0.0)	**<0.001**
Plasma exchanges	2 (3.2)	0 (0.0)	2 (20.0)	0 (0.0)	0 (0.0)	**0.034**
Mortality rate (%)	/	2 (7.1)	2 (20.0)	0 (0.0)	0 (0.0)	0.180

The bold values represent statistically significant P-values (P<0.05).

Due to inflammatory conditions such as primary diseases or secondary skin infections, a large portion of patients with SCARs received alternative antibiotic treatment (n=27, 42.96%) (**Figure 4C**). Among them, 9 cases of cephalosporins-associated SCARs further received lincoamides (4), penicillins (2), quinolones (2), and tetracyclines (1) as alternative antibiotic treatment. Six cases of penicillins-induced SCARs were further treated with cephalosporins (2), quinolones (1), lincoamides (1), cephamicins (1), and carbapenems (1) as alternative antibiotics, respectively. For the 6 SCARs cases caused by nitroimidazoles, 3 were tolerant to lincosamides, while the remaining 3 were tolerant to penicillins, quinolones, and macrolides, respectively. In addition, regarding the 5 cases of quinolones -related SCARs, 3 received lincosamides replacement, and the other 2 received cephalosporins and tetracyclines substitution therapy, respectively. For the 2 cases of macrolides-caused SCARs, cephalosporins and quinolones were subsequently selected as the alternative antibiotics. One tetracyclines-induced case then received macrolides treatment, and 1 lincosamides-induced case was tolerant to cephalosporins.

The mortality was predicted according to the SCORTEN score: 3.2% (0–1 point); 12.1% (2 points); 35.3% (3 points); 58.3% (4 points); and 90.0% (≥5 points) (12). The estimated mortality rate for SJS/SJS-TEN overlap patients was 4.5%, which was not significantly different from the observed mortality rate (7.1%) (*P*=1.000). The complications and cause of death of the 2 SJS patients both included pulmonary infections, with one patient accompanying cancer, which was also a high-risk factor (**Table 5**). The predicted mortality of patients with TEN was 26.1%, whereas the observed mortality rate was found to be 20.0%. There was no significant difference between the predicted and observed mortality rate (*P*=0.672). The 2 deceased patients with TEN all received corticosteroids and IVIG treatment. One patient with ANCA-associated vasculitis and renal insufficiency underwent 10 rounds of hemodialysis, while another one received ICU care due to severe multiple complications, such as pneumonia, sepsis, acute respiratory failure, septic shock, and acute renal failure. By comparing the survival and death of SJS/TEN patients (**Table 6**), it was found that advanced age, cancer, and pneumonia might be risk factors for mortality, and patients could be benefit from longer hospital stays (*P*<0.05). In addition, the median latency of the survival group was shorter than that of the death group (*P*<0.05).

**Table 5:** Decreased cases of SJS/TEN.

Phenotypes	SCORTEN score	Age,y/ Sex	Underlying disease	Causative Antibiotics	Indication for Antibiotics	Incubation period (d)	ALDEN score	Lethal Complications and cause of death	Corticosteroid therapy	IVIG usage
SJS	2	52/F	Cancer	Cefuroxime	Cold	3	3	Pulmonary infection; Hepatic cyst; Cancer	Methylprednisolone (60mg/d*5d+40mg/d*5d)	/
	1	84/F	Hypertension; Coronary heart disease	Cefotaxime	Cold	3	2	Pulmonary infection; Heart involvement	Dexamethasone (12.5mg/d*5d+10mg/d*3d+7.5mg/d*2d)	/
TEN	3	69/M	Asthma;Psoriasis	Metronidazole	Toothache	1	2	Pneumonia; Sepsis; Acute respiratory failure; Septic shock; Acute renal failure; Cardiac arrest; Hypoxic Ischemic Encephalopathy	Dexamethasone (12.5mg/d*7d)	IVIG 25g/d*5d, 10g/d*2d
	5	67/M	ANCA-associated vasculitis; Renal insufficiency	Moxifloxacin	Respiratory infections	5	4	ANCA-associated vasculitis; GI-bleeding; Renal failure; Heart involvement	Methylprednisolone (40mg/d*9d); Dexamethasone (12.5mg/d*13d+10mg/d*9d)	IVIG 15g/d*3d

**Table 6:** Comparison of the survived and deceased patients with SJS/TEN.

Characteristics	Survived (n=34)	Deceased (n=4)	*P* value
Age, y, Median(IQR)	45.0 (37.3-63.8)	68.0 (63.3-72.8)	**<0.001**
Sex, Male	14 (41.2)	2 (50.0)	1.000
Underlying disease			
Cancer	0 (0.0)	2 (50.0)	**0.009**
Liver disease	3 (8.8)	0 (0.0)	1.000
Chronic kidney disease	2 (5.9)	1 (25.0)	0.291
Prior antibiotic allergies	9 (26.5)	0 (0.0)	0.554
Complications			
Pneumonia	10 (29.4)	4 (100.0)	**0.014**
Liver dysfunction	20 (58.8)	1 (25.0)	0.307
Renal dysfunction	11 (32.4)	2 (50.0)	0.595
GI bleeding	3 (8.8)	2 (50.0)	0.076
Heart involvement	9 (26.5)	2 (50.0)	0.564
Sepsis	2(5.9)	1 (25.0)	0.345
Latent period, d, Median(IQR)	2.0 (2.0-4.0)	3.0 (2.5-3.5)	**0.043**
Length of hospital stay, d, Median(IQR)	16.0 (10.0-21.0)	12.0 (10.5-21.5)	**0.018**

The bold values represent statistically significant P-values (P<0.05).

## 4 Discussion

Antibiotic-related SCARs are closely related to significant mortality rates and pose a serious threat to global public health, resulting in an enormous medical burden (31). Although findings of drug-related SCARs have been gradually reported, mainly include case reports or small-scale studies, there is a lack of research specifically focusing on antibiotic-related SCARs (32, 33). In this study, we evaluated 63 cases of antibiotic-induced SCARs targeting Chinese populations and presented a list of culprit antibiotics at the regional level. A comprehensive assessment of the epidemiology, clinical characteristics, treatment, and prognosis of SCARs was also conducted.

*β*-Lactams, especially penicillins and cephalosporins, as well as sulfonamides, glycopeptides and quinolones were generally suspected to be the predominant triggers of SCARs (23, 32, 34), which was consistent with strong antibiotic-associated SCARs signals mined based on the FAERS database (35). Notably, there seems to be varying degrees of differences in the classes and proportion of the main culprit antibiotics in studies based on different regions. A systematic review and meta-analysis involving 2917 patients from over 20 countries also showed that sulfonamides (32%), penicillins (22%), cephalosporins (11%) and quinolones (4%) are considered the main risks related to antibiotic-induced SJS/TEN cases (36). According to a report on the Taiwan population, penicillins (36.7%) and cephalosporins (24.5%) were identified as the main culprit drugs responsible for SJS/TEN and AGEP, whereas glycopeptides (48%) were the main leading cause of DRESS (33). Additionally, besides penicillins (27.4%) and cephalosporins (16.1%), exposure to glycopeptides (12.9%) and sulfonamides (12.9%) were also found to be closely related to antibiotic-induced SCARs in a tertiary referral center in Australia (32). Specifically, in our study, cephalosporins (31.7%), penicillins (25.4%), and quinolones (19.0%) were found to be the leading causes of SCARs, which was consistent with many research findings. However, sulfonamides and glycopeptides were not observed in our study, which was consistent with a retrospective comparative study that included 88 Chinese SJS/TEN patients (37).

The differences may be related to factors such as changes in consumption of antibiotics, regional differences in prescription habits, and genetic variations. As reported in a spatial modelling study estimating antibiotic consumption and usage in 204 countries between 2000 and 2018, global consumption of *β*- lactam antibiotics and quinolones continued to increase, of which penicillins have remained the highest since 2000. However, consumption of sulfonamides and trimethoprim have been decreasing (38). Moreover, there are significant differences in consumption of antibiotics and the proportion of antibiotic categories in different geographical contexts (38). Due to the regional clinical guidelines, the prescription habits might vary among diverse regions. In addition, the complex nature of HLA allele involvement in the pathogenesis of antibiotic-induced SCARs may lead to geographical distribution differences, such as HLA-B*38:02 in Taiwan, HLA-B*15:02 and HLA-C*08:01 in Thailand, and HLA-B*38:01 as well as HLA-B*38:02 for those of European descent, which are significantly associated with sulfamethoxazole/cotrimoxazole-induced SCARs (39). Therefore, considering multiple factors, there may be divergence in the categories and proportions of the main culprit antibiotics of SCARs in different regions.

In addition, cases of exposure to amoxicillin (17.5%) was the most frequently observed, possibly because amoxicillin is one of the most widely used penicillins in clinical practice (40). Moreover, levofloxacin, metronidazole, and cefixime were also accounted for high proportions of 14.3%, 7.9% and 6.3%, respectively, which could be attributed to the primary infection status of patients. It is worth mentioning that the current assessment of drug causality mainly relies on ALDEN score (2) or Naranjo score (30). But when multiple drugs were used simultaneously, it is difficult to choose a suspected drug. In our study, there were 3 cases where antibiotics were used in combination with other drugs owning the same score, making it difficult to determine a culprit drug.

Effective prevention, identification, and termination of suspicious drugs, as well as subsequent antibiotic replacement therapy are particularly important for the management of SCARs. Before using antibiotics for treatment, it is necessary to inquire about the patient’s allergy history. Especially, patients who have not received treatment with penicillins, skin testing is also required. In our study, 13 patients (20.6%) have a history of antibiotic allergy. However, there were still 3 patients who developed SCARs due to the usage of the same allergenic antibiotic, posing an unnecessary threat. Therefore, antibiotic allergy labeling is crucial for the early warning of antibiotic usage for SCARs patients. Additionally, evidence has suggested a significant correlation between the polymorphism of HLA and drug-related SCARs (41, 42), such as the HLA-DRB3*02:02 allele for delayed hypersensitivity to penicillin (43) and the HLA-B*13-01 allele for cotrimoxazole-induced DRESS (44). Therefore, HLA alleles might be developed as effective genetic markers for assessing risk and preventing the antibiotic-related SCARs.

Selecting substitute antibiotics with different structures is crucial for avoiding recurrence or further deterioration of SCARs. Research has shown that antibiotics with similar chemical structures might have a higher risk of cross reactivity. Cross reactivity exists in the first-generation cephalosporins and penicillins, while the second- and third- generation cephalosporins show lower cross reactivity with penicillins (45). Thus, it is relatively safe for patients with penicillin allergy to use second-generation or third-generation cephalosporins, while caution should be exercised when using first-generation cephalosporins (46, 47). Additionally, the cross reactivity between carbapenems and penicillins or cephalosporins is relatively low (≤ 1%), which could also be used as alternative treatment for penicillin or cephalosporin allergies (48). In our study, 27 SCARs patients (42.9%) accepted alternative treatment using antibiotics with structures different from causative drugs and showed tolerance to these antibiotics. Among them, lincomamides occupied the highest proportion (36.7%) as alternative treatments for patients with allergies to other antibiotics, followed by quinolones (16.7%) and cephalosporins (16.7%), which provided references for selecting alternative anti infection strategies in SCARs patients.

There are certain differences in clinical characteristics among different phenotypes of SCARs. In contrast to the SJS/SJS-TEN overlap group, the TEN group seemed to be more prone to pneumonia and liver/kidney/heart dysfunction, which might be related to the relatively poor prognosis of the TEN group. The proportion of mucosal involvement in the SJS/TEN group was significantly higher than that in the DRESS and AGEP groups, which was in concurrence with the reported literature (49). However, the DRESS and AGEP groups were more susceptible to hematologic abnormalities compared with other groups. Liver dysfunction was the most obvious organ damage in the DRESS group, followed by lung and kidney dysfunction, consistent with previous findings (16, 50). Additionally, a high incidence of hypoproteinemia was observed in the DRESS and TEN cohorts, which might increase the incidence of infections and affect the therapeutic effects of drugs. Patients with lower albumin levels usually have more severe conditions, and IVIG is usually chosen as a supplementary treatment in clinical practice (51). According to reports, the presence of malignant tumors, connective tissue diseases, as well as liver and kidney diseases are significantly associated with mortality (37). Apart from that, renal failure, bacterial infection, and epilepsy could also serve as predictive factors for mortality (52). Our research indicated that advanced age, the presence of tumors and lung diseases are risk factors for mortality. Sepsis is also one of the important factors leading to the death of SCARs patients, mostly caused by potential infections before the onset of SCARs (33). Our study showed that 3 SJS/TEN patients were diagnosed with sepsis, and 1 TEN patient with sepsis died. However, there was no significant difference in the proportion of sepsis between the death and the survival group (*P*=0.345), which might be attributed to effective treatment during hospitalization.

Due to the complex pathogenesis, there is currently no specific treatment therapy for SCARs. The application of systemic corticosteroids appears to be a commonly recognized therapeutic approach. Although some studies have indicated that systemic corticosteroids have certain benefits in controlling and improving clinical manifestations (53), their clinical usage is still controversial. Systemic corticosteroid therapy combined with IVIG treatment has also been reported to be effective in improving the clinical outcomes of severe cases (37). Nevertheless, 80.0% of TEN patients and 28.6% of SJS/SJS-TEN overlap patients in our study received the combination therapy, but we did not observe the effect of IVIG on improving their clinical manifestations or outcomes. This result was consistent with a EuroSCAR study on French and German populations, which found that corticosteroids and IVIG had no benefits in reducing the mortality rates of SJS/TEN (5). Thus, further trials are needed to evaluate the potential effects of corticosteroids and IVIG on the clinical courses and outcomes of patients with SCARs. Moreover, a cohort study showed that the usage of cyclosporine might reduce the mortality rate of SJS/TEN (54). A single center retrospective study also showed that cyclosporine therapy might be more beneficial for treating SJS/TEN compared to IVIG usage (55). However, in our study, none of the SJS/TEN patients received cyclosporine treatment, apart from 2 DRESS and AGEP patients. Further exploration is still needed to investigate the impact of cyclosporine on the survival outcomes of SJS/TEN patients.

Owing to the complexity and high risk of SCARs, a larger global network platform is expected to facilitate comprehensive analysis of the epidemiology of SCARs among different populations, further enhancing treatment interventions. It is also crucial to apply immunogenetics and pharmacogenomics to precision medicine in the future, with the potential to identify specific risk populations, thereby achieving optimal patient stratification and individual treatment methods.

## 5 Conclusion

Cephalosporins, penicillins, and quinolones were the most common triggers for SCARs. In particular, amoxicillin, levofloxacin, metronidazole, and cefixime seemed to be the most frequent culprit drugs. The identification of the culprit antibiotics and the use of antibiotics with different structures for alternative therapy are crucial for the management of SCARs. In this study, no benefits were observed for the combined treatment of corticosteroids and IVIG in reducing mortality in SJS/TEN patients. If combined with high-risk factors such as advanced age, cancer, and pneumonia, SJS/TEN patients might be more at risk for mortality.

## Data availability statement

The original contributions presented in the study are included in the article/**Supplementary Material**. Further inquiries can be directed to the corresponding author.

## Ethics statement

The studies involving humans were approved by The ethical committee of Chengdu Second People’s Hospital. The studies were conducted in accordance with the local legislation and institutional requirements. Written informed consent for participation was not required from the participants or the participants’ legal guardians/next of kin in accordance with the national legislation and institutional requirements. Written informed consent was not obtained from the individual(s) for the publication of any potentially identifiable images or data included in this article because the article does not involve images or personal privacy.

## Author contributions

YL: Conceptualization, Methodology, Writing – original draft, Writing – review & editing. LuZ: Data curation, Methodology, Writing – original draft, Writing – review & editing. YZo: Investigation, Software, Writing – original draft. HW: Software, Validation, Writing – original draft. YZh: Resources, Writing – original draft. XG: Formal Analysis, Writing – original draft. QL: Visualization, Writing – review & editing. YY: Supervision, Writing – review & editing. LiZ: Visualization, Writing – review & editing.
