# Supplementary material for: Antibiotic-induced severe cutaneous adverse reactions: a single-center retrospective study over ten years
Source: Front Immunol. 2024 Jul 18;15:1415830. doi: 10.3389/fimmu.2024.1415830 (PMC11291224; doi:10.3389/fimmu.2024.1415830)
Supplement: Supplementary file 1 [file Table_1.doc]

| Antibiotics |  | Phenotypes, n | | | |
| --- | --- | --- | --- | --- | --- |
|  | Total | SJS or SJS-TEN overlap | TEN | DRESS | AGEP |
| Cephalosporins | 7 | 2 | 2 (1, 1#) | 2 | 1 |
| Penicillins | 5 | 3 | 1# | 0 | 1* |
| Levofloxacin | 2 | 0 | 1 | 0 | 1* |
| Metronidazole | 1 | 1 | 0 | 0 | 0 |

Table S1. Different phenotypes of patients with prior antibiotic allergies.

^*^The patient with AGEP showed allergy to both cephalosporins and levofloxacin.

^#^ The patient with TEN was allergic to both cephalosporins and penicillins.

Table S2. Indications for antibiotics in this study.

| Indications for antibiotics |  | Phenotypes, n | | | |
| --- | --- | --- | --- | --- | --- |
|  | Total  (n=63) | SJS or SJS-TEN overlap (n=28) | TEN (n=10) | DRESS (n=7) | AGEP (n=18) |
| Cold | 24(38.1) | 7 | 2 | 3 | 12 |
| Toothache | 7(11.1) | 6 | 1 | 0 | 0 |
| Pharyngitis | 5(7.9) | 3 | 1 | 0 | 1 |
| Pulmonary infections | 5(7.9) | 1 | 2 | 1 | 1 |
| Conjunctivitis | 4(6.3) | 3 | 1 | 0 | 0 |
| Gastritis | 3(4.8) | 2 | 1 | 0 | 0 |
| Tonsillitis | 3(4.8) | 1 | 0 | 1 | 1 |
| Traumatic infection | 1(1.6) | 1 | 0 | 0 | 0 |
| Pyelonephritis | 1(1.6) | 1 | 0 | 0 | 0 |
| Others | 10(15.9) | 4 | 2 | 2 | 4 |
